# Assessment of knowledge, attitude and practice of post exposure prophylaxis for HIV among nurses at Jigme Dorji Wanghuck National Referral Hospital, Bhutan

**DOI:** 10.1371/journal.pone.0238069

**Published:** 2020-08-28

**Authors:** Kezang Tshering, Kinzang Wangchuk, Zimba Letho

**Affiliations:** 1 Department of Pharmacy, Jigme Dorji Wangchuck National Referral Hospital, Thimphu, Bhutan; 2 Department of Community Health, Jigme Dorji Wangchuck National Referral Hospital, Thimphu, Bhutan; 3 Department of Psychiatry, Jigme Dorji Wangchuck National Referral Hospital, Thimphu, Bhutan; Universiteit Maastricht, NETHERLANDS

## Abstract

Nurses are managing huge number of patients infected with human immunodeficiency virus (HIV), which made them highly vulnerable to HIV infection through occupational exposure such as needle stick injuries and splashing of blood/bodily fluids on mucosal surface. This made the practice of post exposure prophylaxis (PEP) for HIV crucial among nurses. Therefore, our study aimed to assess knowledge, attitude and practice of PEP for HIV among nurses in Bhutan. A cross-sectional study was conducted among 221 registered nurses working at Jigme Dorji Wangchuck National Referral Hospital, Bhutan between April and June 2017. A structured self-administered questionnaire was used to collect data and analysed using SPSS version 21. Majority (80.1%) of our participants had poor knowledge regarding PEP for HIV. Although half (51.1%) of our participants had heard about PEP, only 3 (1.4%) attended a formal training on PEP for HIV. However, a significant proportion of nurses (92.3%) had positive attitude towards PEP for HIV. Out of 221 respondents, 95(43%) had been exposed to needle stick injuries and splashing of blood/bodily fluids while managing patients. Despite significant number of exposures, only 2 (2.1%) of them took PEP and completed 28 days of prophylaxis. Lack of protective barriers at work place (56.8%) and poor knowledge on personal protective equipment (14.7%) were major perceived causes of exposure among study participants. No PEP service (30.2%) and lack of support to report incidents (22.6%) were two major reasons leading to failure of PEP practice among exposed individuals. Despite positive attitude exhibited by majority of our respondents, the level of knowledge and practice of PEP for HIV among nurses was very low. Therefore, a formal training on PEP and 24 hours accessible PEP service with proper guidelines are recommended to improve the overall knowledge and practice of PEP against HIV among nurses.

## Introduction

Hundreds of healthcare professionals (HCPs) are infected with human immunodeficiency virus (HIV) as a result of occupational exposure to needle stick injuries each year [1]. According to WHO, more than 3 million percutaneous occupational injuries occur annually among HCPs across the globe [2]. The risk of HIV acquisition through percutaneous exposure to HIV infected blood and mucous membrane exposure were reported as 0.3% [3] and 0.09% [4] respectively.

Post exposure prophylaxis (PEP) involves administering a short course of antiretroviral therapy (ART) following events with high risk of exposure to HIV [5]. Administering antiretroviral agent as a prophylaxis reduces the risk of acquiring HIV infection by 81% after percutaneous exposure [6]. The overall PEP against HIV infection includes first aid after exposure, counselling, risk assessment, laboratory investigations along with consent from source and exposed individuals followed by 28 days of ART and monitoring [7]. The risk of acquiring HIV by HCPs through occupational exposure remained high among Bhutanese HCPs reflecting the notable increase in HIV cases in Bhutan recently. Out of 470 cases reported by the end of 2015, 80% of them were reported between 2007 and 2015 [8]. Moreover, the increased risk of HIV infection through occupational exposures was reported from developing countries [9,10].

Among HCPs, higher rates of percutaneous and mucous membrane exposure to blood and body fluids were observed among nurses [11,12]. Thus, the risk of HIV transmission among nurses through occupational exposure was found to be high. However, there is a paucity of data regarding knowledge, attitude and practice of PEP among nurses in Bhutan. Therefore, our study aimed to assess knowledge, attitude and practice of post-exposure prophylaxis among nurses working at Jigme Dorji Wangchuck National Referral Hospital (JDWNRH), Bhutan.

## Methods

### Study design and setting

A cross-sectional study was conducted from April 15^th^ to June 30^th^, 2017 among registered nurses at Jigme Dorji Wangchuck National Referral Hospital (JDWNRH). JDWNRH is the largest and only tertiary care hospital in Bhutan with 350 bed capacity and highest number of nurses working in Bhutan.

### Sample size calculation and sampling technique

The sample size was calculated using the single proportion formula (n = [Zα / 2] 2P (1-P) / d2) at 95% confidence interval, where Zα / 2 = 1.96, P = 50% prevalence since there was no similar study conducted previously in the study area and d = 5% of marginal. Using above formula, we obtained 384 + 38 (10% dropouts) = 422 as our sample size. Since our exact population of respondents was less than 10,000, we used correction formula (nf = [ni / 1 + ni / N], where nf = minimum required sample size, ni = reduced sample size and N = total number of our respondents. Using correction formula (422 / 1 + 422 / 412), a minimum required sample size of 209 was obtained. All nurses registered under the Bhutan medical and health council as a permanent staff and those involved in direct patient care were approached at their respective departments and asked to participate. Questionnaire was made available at nurse’s station and they were asked to complete questionnaire depending on their convenience. A total of 221 nurses from various departments completed the questionnaire.

### Data collection

Data was collected using a structured self-administered questionnaire developed by researchers based on published studies [13–15]. Questionnaire consisted of socio-demographic characteristics, and questions to assess knowledge, attitude and practice of PEP for HIV infection. Eight questions to evaluate knowledge on PEP included indications of PEP; first aid measures following exposure; preferable time to take PEP after exposure; duration of PEP; effectiveness of PEP; up to how long PEP should be considered after exposure; first line ARV drugs and if they were aware of the hospital policy on PEP. Questions to assess participants sources of information on PEP and if they attended any seminar/training on PEP were also included. To assess attitude towards PEP, seven questions with the response of Yes/No were used. Practice questions included if participants had exposure to HIV risky conditions; perceived cause of exposure; took PEP after exposure; reasons why exposed individuals failed to take PEP; if they completed PEP and if the sources were screened for HIV. Questionnaires were used in English language and pretested among 15% of total participants which were not included in the final analysis.

### Scoring of knowledge, attitude and practice question

Eight questions assessed knowledge of participants on PEP and those who scored ≥75% (≥6 correct response) were considered to have “Good knowledge”, those who scored 50–74% (4–5 correct response) as having “Average knowledge” while participants who scored <50% (≤3 correct response) were categorized as having “Poor knowledge”. Seven questions were used to evaluate participants attitude towards PEP and those who scored 70% and above were categorized as “Positive attitude”. Participant’s practice of PEP was simply assessed if respondents took PEP (antiretroviral therapy) following occupational exposure to high risk conditions.

### Data analysis

Data was checked for completeness, entered, coded and analysed using Statistical Package for Social Sciences (SPSS) version 21. Descriptive statistics (frequency, percentage, mean and standard deviations) were used to present results.

### Ethical consideration

Research Ethics Board of Health (REBH), Ministry of Health of Bhutan approved the study (PO/2017/011) and administrative clearance was obtained from Jigme Dorji Wangchuck National Referral Hospital. The purpose of the study was explained and written informed consent was obtained for voluntary participation from every participant. Personal identifiers were removed and confidentiality of study participants were fully protected.

## Results

### Sociodemographic characteristics

A total of 221 nurses completed the questionnaire and 125 (56.6%) were females. The mean age of the participant was 28.26±5 years (range: 22–42 years), with majority (44.1%) of them between 26–30 years. Most (67.4%) of the nurses had maximum qualification of Diploma in general nursing. Majority (70.1%) of our participants had worked in the hospital for a period of 1–4 years and more than half (55.7%) were working in wards as shown in Table 1.

**Table 1:** Sociodemographic characteristics of nurses in JDWNRH, Bhutan 2017 (N = 221).

Variables	Categories	N (%)
**Sex**	Male	96 (43.4)
	Female	125 (56.6)
**Age**	22–25 years	76 (34.4)
	26–30 years	97 (43.9))
	≥31 years	48 (21.7)
**Education level**	Masters and above	7 (3.2)
	Degree	65 (29.4)
	Diploma	149 (67.4)
**Length of service in hospital**	<5 years	155 (70.1)
	5–9 years	35 (15.8)
	≥ 10 years	31 (14.0)
**Place of work**	Wards	123 (55.7)
	Operation room	13 (5.9)
	Birthing centre	27 (12.2)
	Intensive care units (ICUs)	39 (17.6)
	Ambulatory	19 (8.6)

### Knowledge about PEP among nurses

One hundred and thirteen (51.1%) of our respondents had heard about PEP against HIV. The main sources of information regarding PEP were from college (26.5%) and colleagues/seniors (22.1%) as depicted in Table 2. Thirty-one (27.4%) participants couldn’t remember their source of information and only 3 (1.4%) respondents attended formal training/seminar on PEP for HIV. Majority (77.8%) of our respondents failed to identify indications of PEP and more than half (60.6%) were unaware of appropriate first aid measures following needle stick injury. Sixty-four (29%) participants knew PEP should be initiated within one hour after exposure and 23.5% participants knew PEP should be considered up to 72 hours after exposure. Thirty (13.6%), thirty-nine (17.6%) and seventeen (7.7%) participants knew the correct duration of PEP, effectiveness of PEP and about hospital policy regarding PEP respectively. Majority (89.6%) of our participants failed to identify single ARV drug used as prophylaxis against HIV after exposure. Overall, more than two-third (80.1%) of participants in our study had poor knowledge regarding PEP for HIV (Table 3).

**Table 2:** Knowledge about PEP for HIV among nurses in JDWNRH, Bhutan.

Questions	Response	Frequency (%)
**Have you ever heard about PEP**	Yes	113 (51.1)
	No	108 (48.9)
**Source of information**	College	30 (26.5)
	Colleagues/Seniors	25 (22.1)
	Internet/Media	14 (12.4)
	Books/Journals	5 (4.4)
	Seminar/Training	8 (7.1)
	Can’t remember	31 (27.4)
**Have you attended training or seminar on PEP**	Yes	3 (1.4)
	No	218 (98.6)
**Indications of PEP (multiple response accepted)**	Needle stick injuries	69 (31.2)
	Splashing of blood/bodily fluids on mucosal surface	23 (10.4)
	Rape	49 (22.2)
	Don’t know	172 (77.8)
**First aid measures following needle stick injury**	Promote active bleeding from the wound	20 (9.1)
	Wash thoroughly with soap and water	67 (30.3)
	Don’t know	134 (60.6)
**PEP should be initiated within 1 hour after exposure**	Yes	64 (29)
	No	157 (71)
**For how long PEP should be considered after exposure**	24 hours	69 (31.2)
	48 hours	100 (45.3)
	72 hours	52 (23.5)
**Duration to take PEP**	2 weeks	90 (40.7)
	4 weeks	30 (13.6)
	8 weeks	101 (45.7)
**Effectiveness of PEP**	100%	21 (9.5)
	80–100%	39 (17.6)
	60–70%	126 (57.0)
	50%	30 (13.6)
	<50%	5 (2.3)
**Anti-retroviral drugs used in PEP (multiple response accepted)**	Tenofovir	19 (8.6)
	Zidovudine	11 (5.0)
	Lamivudine	14 (6.3)
	Don’t know	198 (89.6)
**Aware of the hospital policy on PEP for HIV**	Yes	17 (7.7)
	No	204 (92.3)

**Table 3:** Level of knowledge among nurses on PEP against HIV.

Level of knowledge	Frequency (n)	Percent (%)
**Good (≥75%)**	10	4.5
**Average (50–74%)**	34	15.4
**Poor (<50%)**	177	80.1

### Attitude towards PEP for HIV

Overall, 92.3% of our respondents had positive attitude towards PEP for HIV. More than 90% of our participants agreed that PEP against HIV is important where 91.4% believed in behavioral changes through training. The importance of having PEP guidelines at work place and 24 hours accessible PEP services in hospital were agreed by 92.8% and 81% respectively. Majority (74.7%) believed reporting of needle stick injuries as important and 95% of our participants agreed that the risk of acquiring HIV through occupational exposure could be minimized by practicing PEP.

### Practice of PEP for HIV

Among 221 respondents, 95(43%) of them admitted to have had exposure to high risk conditions as shown in Table 4. Maximum (45.3%) exposures were splashing of blood/body fluids on mucosal surface while 15.8% experienced needle prick injury and 38.9% had both needle prick injuries and splashing of blood/body fluids on mucosal surfaces. Regarding perceived cause of exposure, Majority (56.8%) of exposure occurred due to lack of protective barriers at work place, poor knowledge on personal protection equipment (14.7%), accidental (11.6%) and others (16.8%). Among exposed group, sixty-four (67.4%) of them checked the HIV status of their source and 26 (40.6%) were found to be HIV positive.

**Table 4:** Practice of PEP against HIV among nurses.

Questions	Response	N (%)
Have you ever been exposed to HIV risky conditions	Yes	95 (43)
	No	126 (57)
Type of exposure	Needle prick injury	15 (15.8)
	Splashing of blood/body fluids on mucosal surface	43 (45.3)
	Both needle prick and splashing of blood/body fluids on mucosal surface	37 (38.9)
During which working hours you had the exposure	Morning	32 (33.7)
	Evening	16 (16.8)
	Night	21 (22.1)
	Don’t remember	26 (27.4)
Perceived cause of exposure	Lack of protective barriers at work place	54 (56.8)
	poor knowledge on personal protection equipment	14 (14.7)
	Accidental	11 (11.6)
	Others	16 (16.8)
Did you check the HIV status of the patient from where you had your exposure?	Yes	64 (67.4)
	No	31 (32.6)
HIV status of the source	Positive	26 (40.6)
	Negative	38 (59.4)
Received PEP after exposure	Yes	2 (2.1)
	No	93 (97.9)
Completed the prescribed ARV drugs for PEP	Yes	2 (100)
	No	NA
Reason for not receiving PEP after exposure	Lack of support to report incidents	21 (22.6)
	No PEP services	28 (30.1)
	Worried about the side effects	5 (5.2)
	Not important	9 (9.7)
	Others	32 (34.4)

Out of 95 exposed individuals, only two (2.1%) of them took PEP and both completed the prescribed ARV for 28 days. Some of the reasons why exposed individuals failed to take PEP were; no PEP service (30.2%), lack of support to report incidents (22.6%), PEP is not important (9.7%), worried about side effects (3.2%) and others (34.4%).

## Discussion

Among HCPs, nurses were at higher risk of acquiring HIV infection through needle prick injuries and splashing of blood/body fluids on mucosal surface due to involvement in direct patient care. However, there is a paucity of published data regarding PEP against HIV among nurses in Bhutan. Therefore, this was the first study conducted to assess knowledge, attitude and practice of PEP among nurses working at JDWNH.

In this study, majority (80.1%) of the respondents had poor knowledge regarding PEP against HIV. A similar finding was reported among nurses at health district in Cameroon [14]. On the other hand, our respondent’s knowledge on PEP was lower than nurses at Chitwan district in Nepal [15] and Princess Marina hospital in Gaborone [16]. Such discrepancies could be attributed to demographic characteristics of participants such as qualification, years of experiences and formal training attended.

Significant number (43%) of study participants had exposure to HIV risky conditions. This finding is higher compared with studies conducted among healthcare workers in Eastern Ethiopia (17.2%) [17] and Gondar University hospital (33.8%) [7]. But our finding was lower than the findings reported from Southwest Ethiopia (68.5%) [18] and tertiary care hospitals in South India (74.5%) [19]. The major cause of exposure among our respondents were due to lack of protective barriers at work place. Similar reasons were reported in studies conducted among healthcare workers in Southwest Ethiopia [18] and Malaysia hospitals [20].

Despite significant number of exposures occurred among participants, only 2 (2.1%) of them took PEP and completed 28 days of prophylaxis regimen. Low uptake of PEP in this study was similar to what was reported from tertiary hospital in Nigeria [13]. Number of exposed individuals who took PEP in our study was lower compared to nurses who received PEP after occupational exposures in Cameroon (18.9%) [14]. Poor PEP services in the hospital and lack of support to report exposures were two leading causes which resulted in low uptake of PEP after exposure in this study. Similar findings were reported by healthcare workers from governmental health institution in Southwest Ethiopia [18]. Although majority of source tested negative for HIV in our study, HCPs should understand the pathophysiology that sources being HIV negative dose not rule out the risk of acquiring HIV infection completely. However, clinically significant exposures to HIV positive patient took place among our participants. Hence a careful individual based risk assessment should be performed before considering PEP after potential exposures.

Therefore, we recommend hospital authorities to provide urgent in-service training among nurses at JDWNRH on protocols to access PEP and provide continuous supply of antiretroviral agents for prophylaxis. Moreover, hospital management should supply and enforce the use of personal protective equipment among nurses while providing direct patient care to prevent potential occupational exposures.

Cross-sectional design and non-randomized sampling method were main limitations of this study. However, over half of the nurses working at the hospital from different departments participated in the study. This indicates that the findings are most likely applicable to the whole hospital. Since our study was conducted among nurses working at one particular hospital in Bhutan, our results may not be generalized to other hospitals across the country. In addition, our study failed to show the association between outcomes variables and independent variables.

## Conclusion

Knowledge and practice of post exposure prophylaxis for HIV among nurses working at JDWNRH was very low. Despite significant number of exposure to HIV risky conditions such as needle prick injuries and splashing of blood/bodily fluids on mucosal surfaces occurred among study participants, very few of them took PEP. In addition, there was a lack of support in reporting such incidents and there are no proper PEP services available in the hospital.

Therefore, there is an urgent need to provide a proper training on PEP among nurses at JDWNRH to improve their knowledge towards it. Additionally, we recommend health policy makers to put programs in place to scale up the PEP service at JDWNRH to avoid acquisition of HIV through occupational exposures.

## Supporting information

S1 Questionnaire Questionnaire used in study. (DOCX)

S1 Dataset (XLSX)
