# Supplementary material for: Assessment of knowledge, attitude and practice of post exposure prophylaxis for HIV among nurses at Jigme Dorji Wanghuck National Referral Hospital, Bhutan
Source: PLoS One. 2020 Aug 28;15(8):e0238069. doi: 10.1371/journal.pone.0238069 (PMC7454947; doi:10.1371/journal.pone.0238069)
Supplement: S1 Questionnaire — (DOCX) [file pone.0238069.s001.docx]

**QUESTIONNAIRE**

**TITLE:** Assessment of knowledge, attitude and practice of post exposure prophylaxis for HIV among nurses at Jigme Dorji Wanghuck National Referral Hospital, Bhutan.

**INSTRUCTIONS**

Kindly tick (√) or circle the number that corresponds to your answer. If you make a mistake, please cancel the incorrect option and select the preferred answer.

| **SECTION 1. SOCIO-DEMOGRAPHIC CHARACTERISTICS** | | | |
| --- | --- | --- | --- |
| **A** | **B** | **C** | **D** |
| Q1. | Sex | Male | 1 |
|  |  | Female | 2 |
| Q2. | Age (in years) | Number: …………………………… | |
| Q3. | Education level | Masters and above | 1 |
|  |  | Degree | 2 |
|  |  | Diploma | 3 |
| Q4. | Length of service in hospital | <5 years | 1 |
|  |  | 5-9 years | 2 |
|  |  | ≥ 10 years | 3 |
| Q5. | Place of work | Wards | 1 |
|  |  | Operation room | 2 |
|  |  | Birthing center | 3 |
|  |  | Intensive care units (ICUs) | 4 |
|  |  | Ambulatory | 5 |
| **SECTION 2. KNOWLEDGE ABOUT PEP** | | | |
| Q1. | Have you ever heard about PEP | Yes | 1 |
|  |  | No | 2 |
| Q2. | Source of information | College | 1 |
|  |  | Colleagues/Seniors | 2 |
|  |  | Internet/Media | 3 |
|  |  | Books/Journals | 4 |
|  |  | Seminar/Training | 5 |
|  |  | Can’t remember | 6 |
| Q3. | Have you attended training or seminar on PEP | Yes | 1 |
|  |  | No | 2 |
| Q.4 | Indications of PEP (multiple response accepted) | Needle stick injuries | 1 |
|  |  | Splashing of blood/bodily fluids on mucosal surface | 2 |
|  |  | Rape | 3 |
|  |  | Don’t know | 4 |
| Q5. | First aid measures following needle stick injury | Promote active bleeding from the wound | 1 |
|  |  | Wash thoroughly with soap and water | 2 |
|  |  | Don’t know | 3 |
| Q6. | PEP should be initiated within 1 hour after exposure | Yes | 1 |
|  |  | No | 2 |
| Q7. | For how long PEP should be considered after exposure | 24 hours | 1 |
|  |  | 48 hours | 2 |
|  |  | 72 hours | 3 |
| Q8. | Duration to take PEP | 2 weeks | 1 |
|  |  | 4 weeks | 2 |
|  |  | 8 weeks | 3 |
| Q9. | Effectiveness of PEP | 100% | 1 |
|  |  | 80-100% | 2 |
|  |  | 60-70 % | 3 |
|  |  | 50% | 4 |
|  |  | <50% | 5 |
| Q10. | Anti-retroviral drugs used in PEP (multiple response accepted) | Tenofovir | 1 |
|  |  | Zidovudine | 2 |
|  |  | Lamivudine | 3 |
|  |  | Tenofovir & Zidovudine | 4 |
|  |  | Tenofovir & Lamivudine | 5 |
|  |  | Don’t know | 6 |
| Q11. | Aware of the hospital policy on PEP for HIV | Yes | 1 |
|  |  | No | 2 |
| **SECTION 3. ATTITUDE TOWARDS PEP** | | | |
| Q1. | Do you believe PEP is important | Yes | 1 |
|  |  | No | 2 |
| Q2. | Do you believe that training on PEP can bring behavioral changes | Yes | 1 |
|  |  | No | 2 |
| Q3. | It is important to have PEP guideline at the work place | Yes | 1 |
|  |  | No | 2 |
| Q4. | PEP will reduce the risk of acquiring HIV after occupational exposures | Yes | 1 |
|  |  | No | 2 |
| Q5. | It is important to report needle stick injuries | Yes | 1 |
|  |  | No | 2 |
| Q6. | Do you believe PEP works | Yes | 1 |
|  |  | No | 2 |
| Q7. | There is a need of 24 hours accessible PEP service center in the hospital | Yes | 1 |
|  |  | No | 2 |
| **SECTION 4. PRACTICE OF PEP** | | | |
| Q1. | Have you ever been exposed to HIV risky conditions | Yes | 1 |
|  |  | No | 2 |
| Q2. | Type of exposure | Needle prick injury | 1 |
|  |  | Splashing of blood/body fluids on mucosal surface | 2 |
|  |  | Both needle prick and splashing of blood/body fluids on mucosal surface | 3 |
| Q3. | During which working hours you had the exposure | Morning | 1 |
|  |  | Evening | 2 |
|  |  | Night | 3 |
|  |  | Don’t remember | 4 |
| Q4. | Perceived cause of exposure | Lack of protective barriers at work place | 1 |
|  |  | poor knowledge on personal protection equipment | 2 |
|  |  | Accidental | 3 |
|  |  | Others | 4 |
| Q5. | Did you check the HIV status of the patient from where you had your exposure? | Yes | 1 |
|  |  | No | 2 |
| Q6. | HIV status of the source | Positive | 1 |
|  |  | Negative | 2 |
| Q7. | Received PEP after exposure | Yes | 1 |
|  |  | No | 2 |
| Q8. | Completed the prescribed ARV drugs for PEP | Yes | 1 |
|  |  | No | 2 |
| Q9. | Reason for not receiving PEP after exposure | Lack of support to report incidents | 1 |
|  |  | No PEP services | 2 |
|  |  | Worried about the side effects | 3 |
|  |  | Not important | 4 |
|  |  | Others | 5 |
